# Humoral immunity after second dose of BNT162b2 vaccine in Japanese communities: an observational cross-sectional study, Fukushima Vaccination Community Survey

**DOI:** 10.1038/s41598-022-21797-x

**Published:** 2022-11-07

**Authors:** Yurie Kobashi, Takeshi Kawamura, Yuzo Shimazu, Tianchen Zhao, Akira Sugiyama, Aya Nakayama, Yudai Kaneko, Yoshitaka Nishikawa, Fumiya Omata, Morihito Takita, Chika Yamamoto, Makoto Yoshida, Makoto Kosaka, Anju Murayama, Sota Sugiura, Manato Tanaka, Moe Kawashima, Yuna Uchi, Joji Shindo, Tomoyoshi Oikawa, Kenji Shibuya, Tatsuhiko Kodama, Masaharu Tsubokura

**Affiliations:** 1 Department of Radiation Health Management, Fukushima Medical University School of Medicine, Fukushima City, Fukushima 960-1247 Japan; 2 Department of Internal Medicine, Hirata Central Hospital, Hirata Village, Fukushima 963-8202 Japan; 3 Isotope Science Center, The University of Tokyo, Tokyo, 113-0032 Japan; 4 Laboratory for Systems Biology and Medicine, Research Center for Advanced Science and Technology, The University of Tokyo, Tokyo, 153-8904 Japan; 5 Medical & Biological Laboratories Co., Ltd, Tokyo, 105-0012 Japan; 6 Medical Governance Research Institute, Minato-ku, Tokyo 108-0074 Japan; 7 Japan Red Cross Society Fukushima Hospital, Fukushima City, Fukushima 960-8530 Japan; 8 Shindo Clinic, Minamisoma City, Fukushima 975-0061 Japan; 9 Minamisoma Municipal General Hospital, Minamisoma City, Fukushima 975-0033 Japan; 10 Soma Medical Center of Vaccination for COVID-19, Soma City, Fukushima 976-8601 Japan; 11 Tokyo Foundation for Policy Research, Minato-ku, Tokyo 106-6234 Japan

**Keywords:** Immunology, Microbiology

## Abstract

To reveal waning humoral immunity after second dose BNT162b2 vaccinations in a rural Japanese community and determine factors affecting antibody titers. We aimed to report Immunoglobulin G (IgG) antibody against the SARS-CoV-2 spike (S1) protein levels and neutralizing activity in a large scale community based cohort. Methods: Participants in the observational cross-sectional study received a second dose of vaccination with BNT162b2 (Pfizer/BioNTech) and were not previously infected with COVID-19. Questionnaire-collected data on sex, age, adverse vaccine reactions, and medical history was obtained. Results: Data from 2496 participants revealed that older age groups reached a low antibody titer 90–120 days after the second vaccination. Neutralizing activity decreased with age; 35 (13.3%) of those aged ≥ 80 years had neutralizing activity under the cut-off value. Neutralizing activity > 179 days from the second vaccination was 11.6% compared to that at < 60 days from the second vaccination. Significantly lower IgG antibody titers and neutralizing activity were associated with age, male sex, increased time from second vaccination, smoking, steroids, immunosuppression, and comorbidities. Conclusions: Antibody titer decreased substantially over time. Susceptible populations, older people, men, smokers, steroid users, immunosuppression users, and people with three or more comorbidities may require a special protection strategy.

## Introduction

The Coronavirus disease 2019 (COVID-19) pandemic has achieved global crisis status and impact. Vaccines against severe acute respiratory syndrome coronavirus 2 (SARS-CoV-2) were administered worldwide as a rapid solution. Humoral and cellar immunity markers remain high among previously infected patients^1,2^; thus, immunity after vaccination is expected to remain high. However, reports from USA and Israel have shown that the number of newly infected patients was increasing within a few months of vaccination, especially for delta variants of the virus^3,4^. Antibody titer kinetics differ between post-vaccination and post-infection populations, suggesting that humoral immunity in both needs to be examined^5^. Modelling studies have shown that a low antibody titer is associated with weak protection against infection^6,7^. Similarly, neutralizing antibody levels were lower in those who had breakthrough infections in case–control studies^8^. Therefore, a community survey for humoral immunity after vaccination is crucial in establishing the strategy required to protect the population from SARS-CoV-2.

Aspects of humoral immunity in the post-vaccination population have already been investigated. For instance, the peak antibody titer after two doses of vaccine is lower in susceptible populations^9^. Humoral immunity is reduced, especially in the geriatric population and those on immunosuppressive drugs, at 6 months after vaccination^10^. However, only a few of these studies assessed antibody titers after vaccination in large sample sizes.

Japan experienced a surge in COVID-19 cases in August, 2021; a total of 1,726,823 cases were reported by 27th November. BENT162b2 vaccination was offered beginning March 2021 to health care workers, older adults and people with comorbidities, and then progressively to the general population. A total of 77% of the population aged ≥ 12 years who were eligible for vaccination had received second doses by 27th November. As a result, the number of infected patients decreased in November, and deregulation was being implemented by the government.

By December, the requirement for a third vaccination dose was actively discussed in Japan, as in other countries. However, there was confusion about the population eligible for the third dose. In the beginning, only those who had a second dose over 8 months previously were eligible, but on 16^th^ November, those who had a second dose only 6 months previously were also eligible. This was quickly reverted on 17^th^ November, and the policy returned to the earlier conditions where the third dose was available only for those who had a second dose more than 8 months previously.

Considering these situations, humoral immunity examination after vaccination was required to best formulate a strategy for infection prevention, especially in rural communities where older adults and susceptible populations live. Several studies have attempted to describe the relationship between higher antibody titer and health status in Japan (i.e., medications, comorbidities, alcohol consumption, smoking habits, and adverse reactions after vaccination)^11–13^. However, only a few of these studies mention the kinetics of antibody titers after vaccination. In addition, antibody testing among healthcare workers has been continuously conducted in the Ken-chu District of Fukushima Prefecture since 2020^14,15^. Therefore, this area is suitable for assessing the antibody titers after vaccinations.

We aimed to assess the antibody titers on a large scale from Japanese communities to demonstrate the waning humoral immunity after the second BNT162b2 vaccination in a real-world rural community and to determine the factors affecting antibody titers. This survey was supported by the Japan Agency for Medical Research and Development, and this study presents the data generated from the first of five surveys which will be administered in the same cohort every 3 months.

## Materials and methods

### Study design and population

This was an observational cross-sectional study. The participants, recruited primarily from the rural Fukushima prefecture, included healthcare workers, frontline workers, administrative officers, general residents, and residents of long-term care facilities. Hospital groups and municipalities in Kenchu District and Soso District in Fukushima prefecture cooperated to recruit participants and perform blood sampling. Participants were mainly recruited from Ishikawa gun, Soma city, and Minami-soma city. A total of 1432 participants were recruited from Ishikawa gun, including 473 healthcare workers and 959 non-healthcare workers and general residents. A total of 500 participants were recruited from Soma city, mainly non-healthcare workers and general residents. A total of 594 participants were recruited from Minami Soma city, including 150 healthcare workers and 444 non-healthcare workers and general residents. The study was approved by the ethics committees of Hirata Central Hospital (number 2021-0611-1) and Fukushima Medical University (number 2021-116). This study conforms to The Code of Ethics of the World Medical Association (Declaration of Helsinki).

**Figure 1:**
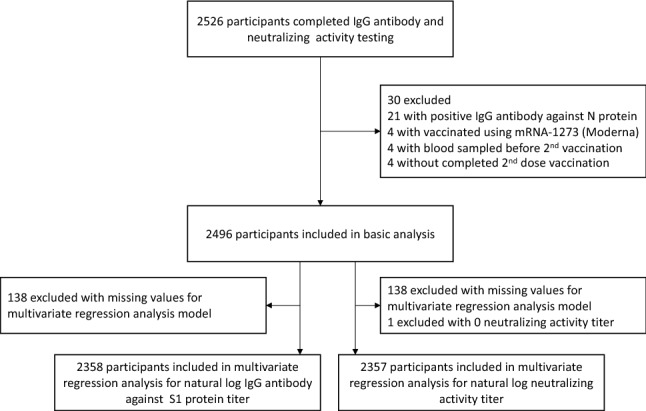
Requirement of participants. The participants were mainly recruited from rural Fukushima prefecture, including health care workers, frontline workers, administrative officers, general residents, and residents of long-term care facilities.

A total of 2526 participants completed the serological testing. The eligibility criteria of study participants were second dose vaccination with BNT162b2 (Pfizer/BioNTech) vaccine, no previous COVID-19 infection, and blood sampling being performed at least 10 days after the second vaccination (Fig. 1). We conducted sequential antibody surveys in 2021, and the peak titers were around 10 days from the second vaccination; thus, we conducted blood sampling after 10 days from the second vaccination^16^. Children ≥ 12 years were allowed to receive the COVID-19 vaccination; thus, we included 65 participants aged 12–19. The official interval between first and second doses was 21 days. A paper-based questionnaire was then conducted for all participants; data on sex, age, daily medications, medical history, date of vaccination, adverse reactions after vaccination, and type of vaccination were retrieved from the questionnaire. Written informed consent was obtained from all participants. All participants were given the serological assay results individually in writing, and authors frequently conducted seminars for medical staff and residents on how to interpret the serological results.

### Study design of the serological assay

This research was supported by the Japan Agency for Medical Research and Development, a national project. All blood sampling was conducted between 8th September and 8th October in each facility in rural Fukushima prefecture. Centrifugal separation of blood samples was conducted in each facility, and serum samples were sent to Tokyo University. All serological assays were performed at Tokyo University, between 22nd September and 28th October. Levels of immunoglobulin G (IgG) antibody against the SARS-CoV-2 spike (S1) protein and the neutralizing activity were measured as the secondary immune status outcomes after second dose vaccination. IgG antibody titers against the SARS-CoV-2N-protein were used to determine the past COVID-19 infection status. We choose IgG(S) because the accuracy to measure hormonal immune response was assured in previous studies^14,15^.

### Serological assay

All serological assays were performed using the CLIA assay with iFlash 3000 (YHLO Biotech, Shenzhen, China) and iFlash-2019-nCoV series (YHLO Biotech, Shenzhen, China) as reagents. The cut-off values for each parameter (IgG antibody against S1 protein, neutralizing activity, and IgG antibody against N protein) were 10 arbitrary units per milliliter (AU/mL), which were the official cut-off values. A neutralizing activity over > 500 AU/mL was not a guarantee of accuracy; thus, we interpreted measures of neutralizing activity > 500 AU/mL as being equal to 500 AU/mL. The testing process complied with the official guidelines. Quality assurance checks were performed every day before starting the measurement. For neutralizing activity, AU/mL × 2.4 was used to convert to International Units (IU/mL). For IgG, AU/mL × 1.0 was used to convert to binding antibody units (BAU/mL).

### Statistical analysis

The median and mean antibody titer values were determined for both ‘age’ and ‘duration since second vaccination’ groups to identify groups with low antibody titers. The number of individuals with antibody titers under the cut-off values was shown group-wise. The mean titer for the ‘duration since second vaccination’ group was adjusted using a country-wide population age distribution based on the Japanese population in 1985. Violin plots were used to show the distribution of natural log-transformed antibody titer. Neutralizing activity > 500 AU/mL was taken as 500 AU/mL in all statistical methods.

Log transformed anti-S1 IgG antibody and neutralizing activity were analyzed as dependent variables with multiple linear regression to determine their association with the antibody titers and the participants’ characteristics. Age, sex, duration since second vaccination, the interval between vaccinations, smoking habits, alcohol consumption, daily medications, comorbidities, and the number of adverse reactions after the second vaccination dose were included in the multiple linear regression as dependent variables. We included comorbidities that are associated with low antibody titers in the literature: hypertension^9^, diabetes^9^, heart disease^9^, kidney disease^17^, cancer^18,19^, connective tissue disease^20^, and immune deficiency^9,21^. We considered the interaction between age and duration since vaccination; however, a relationship was not established. Individuals with missing data on dependent variables were excluded from the regression analysis.. STATA IC (Lightstone, Texas, USA, version 15) was used for all analyses.

## Results

Between 8th September and 8th October, 2526 individuals in a rural area of Fukushima Prefecture completed blood sampling. Single blood sampling was performed on all participants. Of these, 21 individuals were over cut-off values of Anti-N antibody and thus excluded from participating in the study. Finally, 2496 participants were eligible for this study (Fig. 1). There were no missing data for anti-S1 IgG antibody, neutralizing activity, IgG antibody titers against N protein, age, and sex. Of these, 2358 participants (94.5%) without missing data were included in the multiple regression analysis.

The median age was 51 years (IQR; 37–67 years). Demographic characteristics and coexisting conditions are provided in Table 1. A total of 58 individuals were on regular steroids, and 24 were on immunosuppressive therapy (Table 1). The median of the interval between first dose and second dose was 21 days (IQR 21–22) among the whole cohort; of these 98 (3.9%) were < 21 days, 1,722 (69.0%) were 21 days, 464 (18.6%) were 22–28 days, and 212 (8.5%) were > 22 days. The relationships between the age group and the duration since the second vaccination are shown in Fig. S1. A total of 89% of participants who were ≥ 80 years had blood sampled in the period of 90–120 days after the second vaccination dose. The interquartile range and median for both anti-S1 IgG antibody and neutralizing activity titers for each age group are shown using a box plot (Fig. S1). The peak antibody titers were lower among the older age group; older participants reached a low antibody titer, 90–120 days after the second vaccination (Fig. S1).

**Table 1:** Demographic and clinical characteristics of participants.

	< 40 years	40–64 years	> 65 years	Overall
**Sex**				
Male	338 (47.0)	412 (38.0)	279 (40.3)	1029 (41.3)
Female	381 (53.0)	671 (62.0)	413 (59.7)	1465 (58.7)
**Date from 2nd vaccination**				
< 30 days	9 (1.3)	12 (1.1)	1 (0.1)	22 (0.9)
30–89 days	251 (34.9)	379 (35.0)	94 (13.6)	724 (29.0)
≥ 90 days	459 (63.8)	692 (63.9)	597 (86.3)	1748 (70.1)
**Comorbidities**				
Hypertension	8 (1.1)	231 (21.3)	426 (61.6)	665 (26.7)
Dyslipidemia	12 (1.7)	122 (11.3)	141 (20.4)	275 (11.0)
Heart disease	14 (2.0)	47 (4.3)	136 (19.7)	197 (7.9)
Diabetes	6 (0.8)	69 (6.4)	107 (15.5)	182 (7.3)
Allergic disease	69 (9.6)	93 (8.6)	21 (3.0)	183 (7.3)
Asthma	48 (6.7)	44 (4.1)	27 (3.9)	119 (4.8)
Liver disease	11 (1.5)	44 (4.1)	58 (8.4)	113 (4.5)
Cancer	3 (0.4)	35 (3.2)	46 (6.7)	84 (3.4)
Gout	5 (0.7)	43 (4.0)	25 (3.6)	73 (2.9)
Thyroid disease	8 (1.1)	36 (3.3)	11 (1.6)	55 (2.2)
Lung disease	12 (1.7)	11 (1.0)	28 (4.1)	51 (2.0)
Mental disease	17 (2.4)	16 (1.5)	13 (1.9)	46 (1.8)
Rheumatism	2 (0.3)	16 (1.5)	19 (2.8)	37 (1.5)
Kidney disease	6 (0.8)	7 (0.7)	13 (1.9)	26 (1.0)
Anaphylaxis	6 (0.8)	7 (0.7)	5 (0.7)	18 (0.7)
Connective tissue disease	4 (0.6)	6 (0.6)	5 (0.7)	15 (0.6)
Immune deficiency	2 (0.3)	4 (0.4)	0 (0.0)	6 (0.2)
Others	51 (7.1)	146 (13.5)	189 (27.3)	386 (15.5)
**Daily medication**				
Steroids	9 (1.3)	23 (2.1)	26 (3.8)	58 (2.3)
Immunosuppression	6 (0.8)	10 (0.9)	8 (1.2)	24 (1.0)
NSAIDs	31 (4.3)	77 (7.1)	82 (11.9)	190 (7.6)
Acetaminophen	8 (1.1)	22 (2.0)	30 (4.3)	60 (2.4)
Antihistamines	46 (6.4)	63 (5.8)	43 (6.2)	152 (6.1)

NSAIDs, non-steroidal anti-inflammatory drugs.

**Figure 2:**
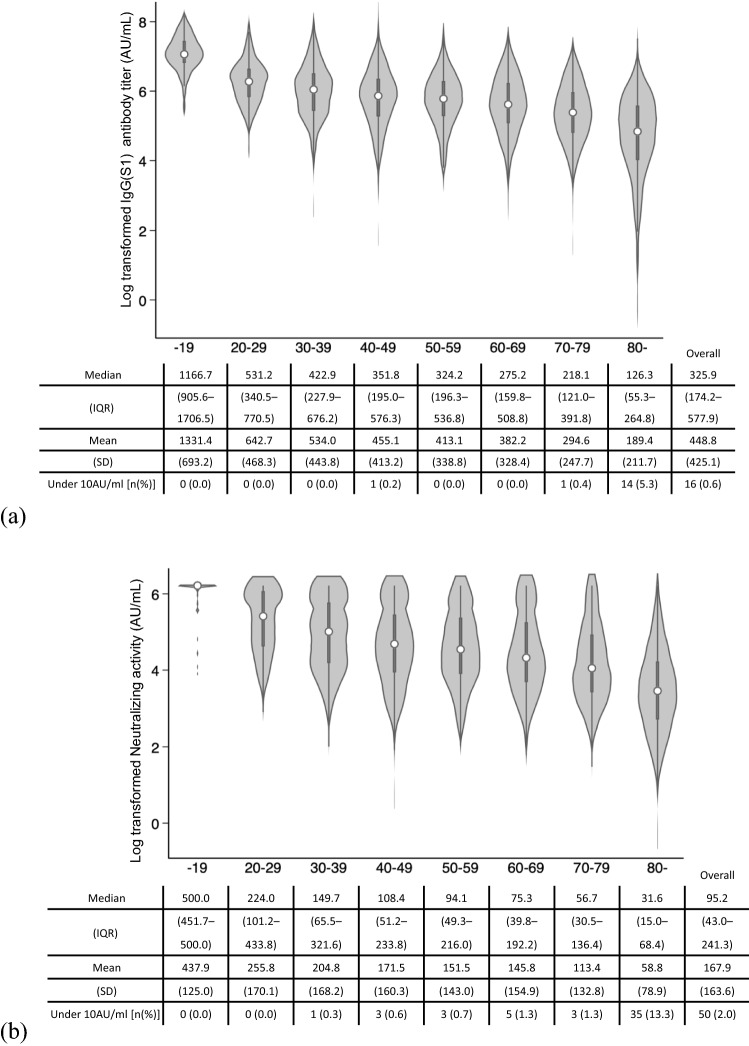
Distribution of (**a)** IgG antibody titer against S1 protein and (**b**) neutralizing activity by age groups. Anti-S1 IgG antibody and neutralizing activity were log-transformed. The cut-off values of each antibody were 10 arbitrary units per milliliter (AU/mL).

Antibody titers were analyzed based on age groups to assess the differences in antibody titers across the demographic characteristics. The median anti-S1 IgG antibody and neutralizing activity titers decreased with age (Fig. 2). The median value of anti-S1 IgG antibody in participants aged ≥ 80 years was 10.9% of the median titer of those aged ≤ 19 years. The number of people with an antibody titer under cut-off value was 16; of these, 14 were aged ≥ 80 years. The median neutralizing activity decreased with age; 35 (13.3%) of those aged ≥ 80 years had neutralizing activity under the cut-off value, which is remarkably higher than in the other age groups. A total of 50 participants had antibody titers below the cut-off; of these, 15 were on steroids, four on immunosuppressants, seven on non-steroidal anti-inflammatory drugs, two on acetaminophen, and nine on antihistamines. Also, among these 50 participants, 28 had hypertension, 13 had heart disease, eight had rheumatism, and eight had diabetes.

**Figure 3:**
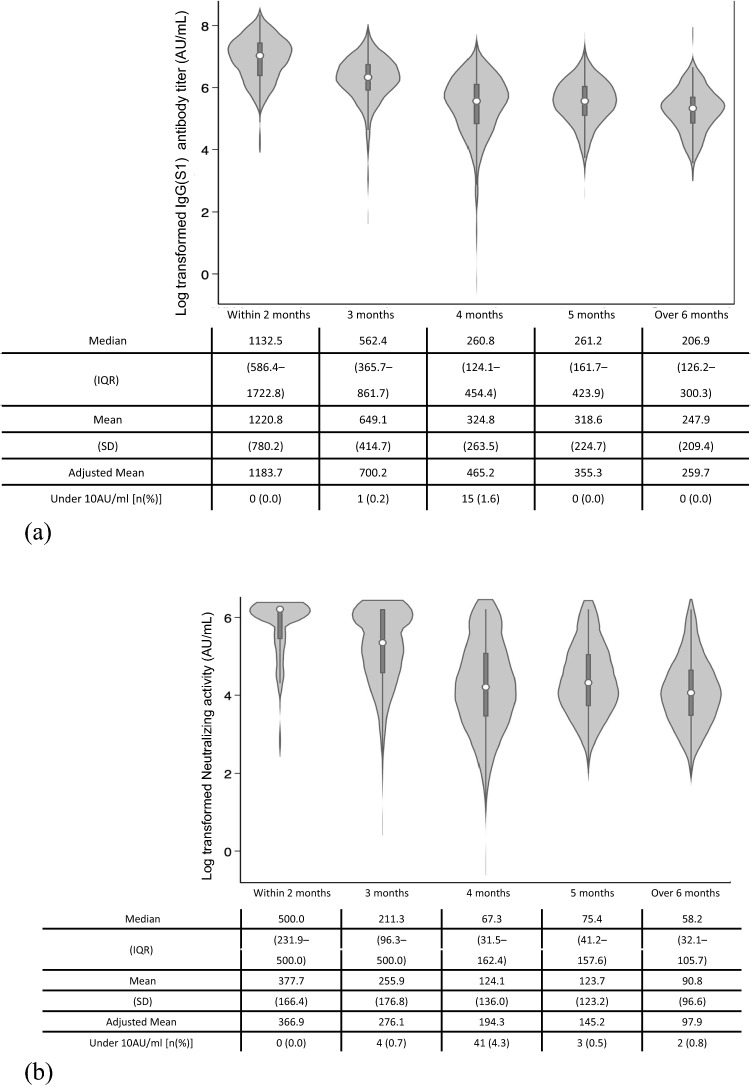
Distribution of (**a**) IgG antibody titer against S1 protein and (**b**) neutralizing activity by groups based on duration from second vaccination. Anti-S1 IgG antibody and neutralizing activity were log-transformed. The cut-off values of each antibody were 10 arbitrary units per milliliter (AU/mL). The adjusted mean titer was calculated using the age distribution of the whole country’s population based on the Japanese model population in 1985 (we do not have the new version of the model population).

Antibody titers and neutralizing activity were assessed relative to the duration since the second vaccine dose (Fig. 3). The median anti-S1 IgG antibody and neutralizing activity decreased as the duration from the second vaccine dose increased. The median anti-S1 IgG antibody levels of participants who had the second dose > 179 days before testing was 18.3% lower than the median in participants who had the second dose < 60 days before testing. The adjusted mean value of anti-S1 IgG antibody levels of participants who had the second dose > 179 days before testing was 21.9% lower than the adjusted mean in participants who had the second dose < 60 days before testing. The adjusted mean value with the Japanese population age structure of 1985 tended to be slightly higher than the actual data. The frequency of levels under the cut-off value was the highest in participants who had the second dose > 4 months before testing (15 participants).

Similarly, neutralizing activity decreased as the duration since the second vaccination increased. The median value of neutralizing activity in participants who had the second dose > 179 days before testing was 11.6% lower than that of participants who had the second dose < 60 days before testing. The adjusted mean value for neutralizing activity for those who had the second dose > 179 days before testing was 26.7% lower than the mean in those who had the second dose < 60 days before testing. The frequency of neutralizing activity under the cut-off value was the highest in participants who had the second dose > 4 months before testing (41 participants).

We used multivariate linear regression analysis to predict the variables that affect the log anti-S1 IgG antibody (Table 2). Significantly lower IgG antibody titer was associated with older age, male sex, longer time since second vaccination, smoking, regular use of steroids and immunosuppressants, and comorbidities (i.e., diabetes, heart disease, and connective tissue disease). Conversely, significantly higher IgG antibody titer was associated with female sex, 22–28 days interval between vaccinations, increased number of adverse reactions at second vaccination (such as > 37.5 ℃ fever, fatigue, headache, joint pain, diarrhea, nausea, and dizziness; Table 2), and hypertension.

**Table 2:** Linear regression analysis of variables associated with IgG antibody titre after BNT162b2.

	B	SE	Beta	P value
Age	− 0.021	0.001	− 0.399	< 0.001
Sex (base: male)	0.082	0.035	0.040	0.019
Date from 2nd vaccination	− 0.014	0.001	− 0.431	< 0.001
**Interval between vaccinations (base: 21 day)**				
< 21 days	0.017	0.082	0.003	0.834
22–28 days	0.130	0.042	0.051	0.002
> 28 days	0.080	0.058	0.022	0.167
**Smoking (base: none)**	− 0.181	0.042	− 0.070	< 0.001
**Alcohol (base: none)**				
Sometimes	− 0.040	0.038	− 0.017	0.300
Everyday	− 0.022	0.046	− 0.008	0.627
**Daily medication (base: none)**				
Steroids	− 0.772	0.117	− 0.111	< 0.001
Immunosuppression	− 0.636	0.178	− 0.060	< 0.001
NSAIDs	− 0.045	0.061	− 0.012	0.459
Acetaminophen	0.001	0.108	0.000	0.990
Antihistamines	− 0.107	0.068	− 0.025	0.115
**Comorbidities (base: none)**				
Hypertension	0.106	0.043	0.047	0.013
Diabetes	− 0.191	0.063	− 0.049	0.003
Heart disease	− 0.132	0.061	− 0.035	0.031
Kidney disease	− 0.209	0.150	− 0.022	0.162
Cancer	0.012	0.086	0.002	0.887
Connective tissue disease	− 0.285	0.123	− 0.040	0.021
Immune deficiency	0.051	0.307	0.003	0.869
**Adverse reaction (base: none)**				
1	0.271	0.044	0.112	< 0.001
2≥	0.311	0.042	0.155	< 0.001

Connective tissue disease included Rheumatism.

Adverse reaction included whole body adverse reaction at 2nd dose (> 37.5 °C fever, fatigue, headache, joint pain, diarrhea, nausea, dizziness, and other).

NSAIDs, non-steroidal anti-inflammatory drugs.

Linear regression analysis was performed to predict the variables that affect the log-transformed neutralizing activity. The results were almost identical to those of the anti-S1 IgG antibody; however, heart disease and hypertension were not significantly associated with the neutralizing activity titer. An interval between vaccinations of > 28 days was significantly associated with higher neutralizing activity (Table 3).

**Table 3:** Linear regression analysis of variables associated with neutralizing activity after BNT162b2.

	B	SE	Beta	P value
Age	− 0.022	0.001	− 0.384	< 0.001
Sex (base: male)	0.108	0.040	0.048	0.007
Date from 2nd vaccination	− 0.014	0.001	− 0.401	< 0.001
**Interval of vaccinations (base: 21 days)**				
< 21 days	− 0.025	0.094	− 0.004	0.794
22–28 days	0.197	0.048	0.069	< 0.001
> 28 days	0.162	0.067	0.040	0.015
**Smoking (base: none)**	− 0.246	0.048	− 0.085	< 0.001
**Alcohol (base: none)**				
Sometimes	− 0.052	0.044	− 0.020	0.238
Everyday	− 0.063	0.053	− 0.021	0.233
**Daily medication (base: none)**				
Steroids	− 0.636	0.136	− 0.082	< 0.001
Immunosuppression	− 0.474	0.209	− 0.039	0.024
NSAIDs	− 0.056	0.071	− 0.013	0.424
Acetaminophen	− 0.077	0.124	− 0.010	0.538
Antihistamines	− 0.103	0.078	− 0.021	0.189
**Comorbidities (base: none)**				
Hypertension	0.070	0.049	0.027	0.157
Diabetes	− 0.211	0.073	− 0.048	0.004
Heart disease	− 0.044	0.070	− 0.011	0.531
Kidney disease	− 0.213	0.173	− 0.020	0.217
Cancer	0.002	0.100	0.000	0.986
Connective tissue disease	− 0.330	0.143	− 0.041	0.021
Immune deficiency	0.197	0.355	0.009	0.578
**Adverse reaction (base: none)**				
1	0.277	0.051	0.102	< 0.001
2≥	0.355	0.048	0.158	< 0.001

Connective tissue disease included Rheumatism.

Adverse reaction included whole body adverse reaction at 2nd dose (> 37.5 °C fever, fatigue, headache, joint pain, diarrhea, nausea, dizziness, and others).

NSAIDs, non-steroidal anti-inflammatory drugs.

## Discussion

Understanding humoral immunity after vaccination in communities is crucial for COVID-19 infection control. This study revealed the waning of antibody titers after the second dose of BNT162b2 in real-world rural communities in Japan and determined the factors associated with decreased antibody titer.

Antibody titers decreased substantially over time in a rural community in Japan. In particular, both anti-S1 IgG antibody and neutralizing activity declined significantly within 3 months and declined slowly thereafter. A similar trend was observed in other cohort studies^10^. This speedy decline in humoral immunity after vaccination is faster than in vaccines for other diseases. Reports from Israel and the USA have shown an increase of infected patients a few months after vaccination^3,4^. For those > 4 months away from vaccination, humoral immunity has decreased notably from its peak.

Antibody titers showed a marked sharp decline among those aged ≥ 80 years. Low levels of both humoral and cellular immunity have been reported among older age groups, especially in people > 80 years^22–24^. Medical literature also reports the efficacy of the third vaccination in the elderly^25^. Stringent infection prevention strategies should be implemented for people ≥ 80 years and people who frequently come into contact with them.

In addition to the older adult population and the population with a relatively long interval since the last vaccination, male sex, smokers, steroid users, immunosuppressant users, individuals with diabetes, and individuals with connective tissue disease had lower antibody titers and neutralizing activities. Those with heart disease had significantly lower IgG titers but not neutralizing activity. Among these multi-risk susceptible populations, long-lasting infection and multi-mutation of virus genes were reported because of the impossibility of virus elimination^26–28^. In addition, patients who suffer breakthrough infections are often immunocompromised^29^. In high-risk populations such as these, monitoring humoral and cellular immunity after vaccination might be required.

The IgG and neutralizing activity titers were higher among those vaccinated with 22 to 28 days intervals between doses than those vaccinated with ≤ 21 days intervals. IgG and neutralizing activity were also higher among those who had experienced a systemic adverse reaction at the second dose. The results are consistent with previous reports that higher antibody titers are associated with longer intervals between vaccinations^11^. A vaccination interval of 22–28 days is the most efficacious in terms of antibody titer in the Japanese population.

Limitations of our study include the fact that the study design was a cross-sectional study with various demographic characteristics within participants, while the purpose of this study was to observe the kinetics of humoral immunity. The sample size was too small for findings to be generalized to the general population. The proportion of females was high. Data on comorbidities and medications were self-reported. Neutralizing activity titers > 500 AU/mL were not accurate and were taken as 500 AU/mL. Cellular immunity was not investigated. Hypertension was significantly associated with higher anti-S1 IgG antibody, but this result was inconsistent with those of other published works. Finally, the blood samples were not obtained from the participants at a fixed time point following the second dose. Therefore, variations between anti-S1 IgG antibody titers are expected. Humoral immunity was lower among older age groups and those with a long duration between vaccination and testing. The kinetics of individual humoral immunity and the factors associated with immunity need to be studied for a more extended period. To clarify these issues, we will continue to survey humoral immunity in the same cohort as a national project.

## Conclusion

This study shows the waning of humoral immunity after the second vaccination. Neutralizing activity under the cut-off value is mainly found among those > 80 years old and > 4 months after the second vaccination. Susceptible populations, older age, males, smokers, steroid users, immunosuppressant users, and people with three or more comorbidities may require a special protective strategy and perhaps monitoring for immunity after vaccination.

## Supplementary Information

Supplementary Information.

## Data Availability

The data that support the findings of this study are available from Fukushima Medical University School of Medicine but restrictions apply to the availability of these data, which were used under license for the current study, and are not publicly available. Data are however available from the corresponding author upon reasonable request and with permission of Fukushima Medical University School of Medicine.
