# Supplementary material for: Humoral immunity after second dose of BNT162b2 vaccine in Japanese communities: an observational cross-sectional study, Fukushima Vaccination Community Survey
Source: Sci Rep. 2022 Nov 7;12:18929. doi: 10.1038/s41598-022-21797-x (PMC9640658; doi:10.1038/s41598-022-21797-x)
Supplement: Supplementary file 1 — Supplementary Information. [file 41598_2022_21797_MOESM1_ESM.pdf]

Supplemental table 1. Date from 2nd vaccination by aging groups

	-19	20-29	30-39	40-49	50-59	60-69	70-79	80-	Overall
Within 2 months	17	19	31	43	24	16	4	2	156
	(26.2)	(7.3)	(7.9)	(8.9)	(5.8)	(4.2)	(1.7)	(0.8)	(6.3)
3 months	35	67	91	131	107	91	45	23	590
	(53.9)	(25.9)	(23.0)	(27.0)	(25.9)	(24.0)	(19.3)	(8.8)	(23.7)
4 months	9	48	80	106	126	198	151	233	951
	(13.9)	(18.5)	(20.3)	(21.9)	(30.4)	(52.1)	(64.8)	(88.6)	(38.1)
5 months	4	92	129	142	96	61	30	5	559
	(6.2)	(35.5)	(32.7)	(29.3)	(23.2)	(16.1)	(12.9)	(1.9)	(22.4)
Over 6 months	0	33	64	63	61	14	3	0	238
	(0.0)	(12.7)	(16.2)	(13.0)	(14.7)	(3.7)	(1.3)	(0.0)	(9.5)

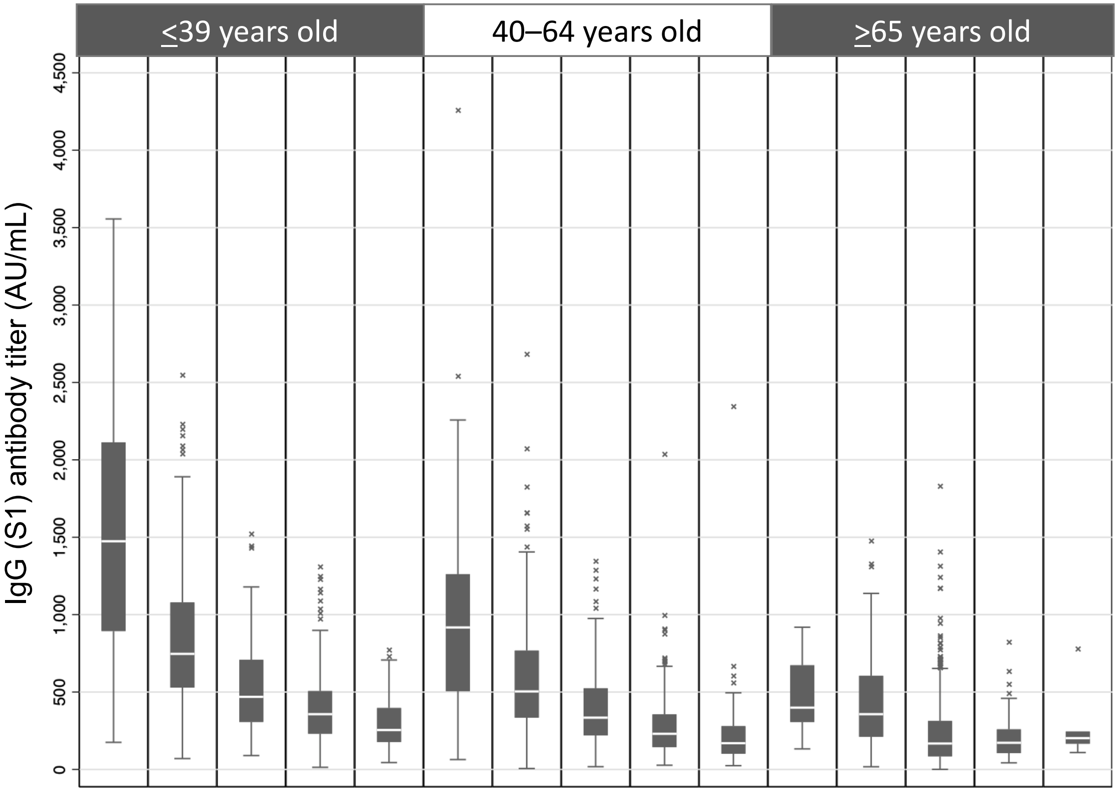

Supplemental figure 1-(a)

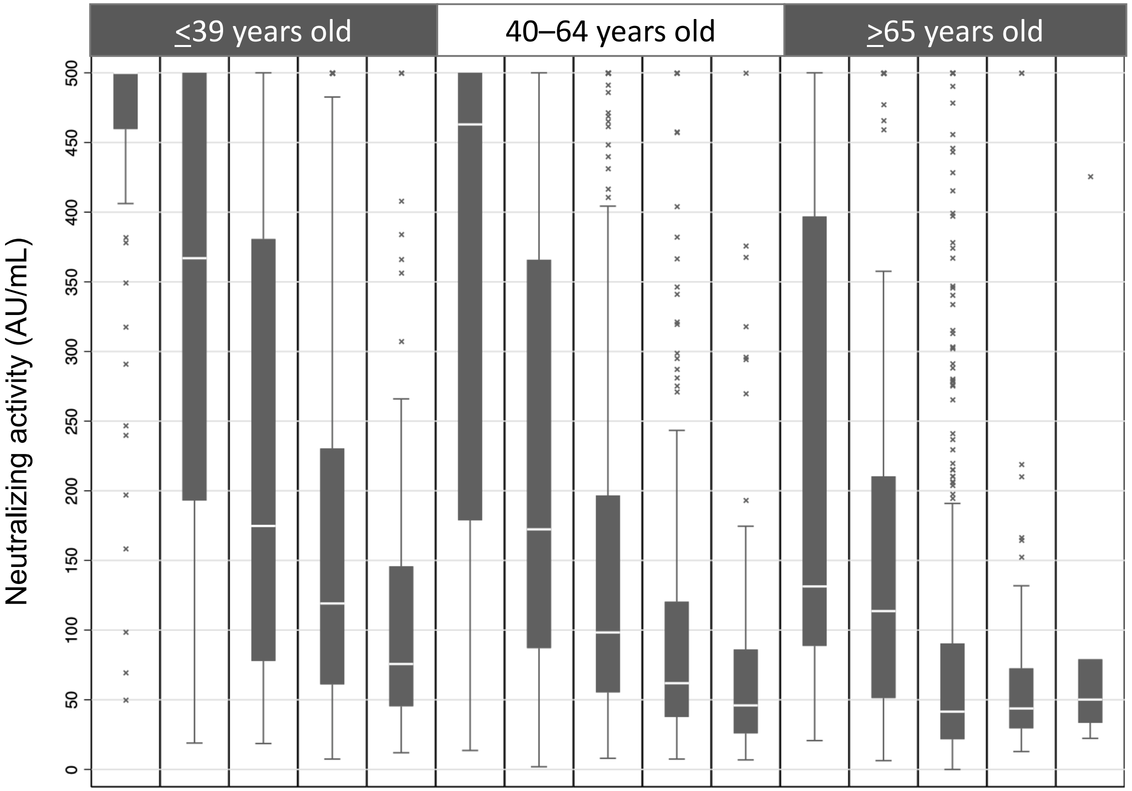

Supplemental figure 1-(b)

Supplemental figure 1. The kinetics of median of (a) IgG antibody titer against S1 protein and (b) neutralizing activity after the second dose of BENT162b2 by each age group

The duration from the second vaccination was classified as within two months, three months, four months, five months, and over six months.
